# Screening of hospital admissions for COVID-19 in Brunei Darussalam

**DOI:** 10.5365/wpsar.2020.11.2.009

**Published:** 2021-04-21

**Authors:** Sanny Zi Lung Choo, Hazirah Shafri, Fatimah Al-Zahara Johan, Norwani Basir, Pui Ling Chong, Muhammad Syafiq Abdullah, Rosmonaliza Asli, Jackson Tan, Dilip Joseph Thottacherry, Mohammad Ady Adillah Ahmad, Vui Heng Chong

**Affiliations:** a Raja Isteri Pengiran Anak Saleha Hospital, Brunei Darussalam.; b Institute of Health Sciences, Pengiran Anak Puteri Rashidah Sa’adatul Bolkiah, Universiti Brunei Darussalam, Brunei Darussalam.; c Suri Seri Begawan Hospital, Brunei Darussalam.; d Pengiran Isteri Hajah Mariam Hospital, Temburong, Brunei Darussalam.

Since late December 2019, an outbreak of coronavirus disease 2019 (COVID-19), caused by severe acute respiratory syndrome coronavirus 2 (SARS-CoV-2), has spread globally, resulting in a pandemic. As of 30 March 2021, 126 million confirmed cases had been reported worldwide, with 2.7 million deaths. (*1*)

Brunei Darussalam reported its first case of COVID-19 on 9 March 2020, and as of 11 April 2021, there were 219 confirmed cases. (*2*) Apart from limited small clusters, Brunei Darussalam remains at WHO Level 2 of COVID-19 transmission with the last documented local infection on 6 May 2020. Several measures were taken by the Government, headed by the Ministry of Health, to prevent or contain community spread. These included active case identification (i.e. screening at points of entry and surveillance in clinics), contact tracing by the Department of Public Health, isolation of confirmed cases in a designated hospital (the National Isolation Centre, Tutong District), limiting public gatherings (closure of schools and places of worship, cancellation of public gatherings and banning of large private functions) and continued advice on physical distancing (through all media). In public and private hospitals and clinics, measures to prevent nosocomial spread of COVID-19 included limiting entry points, with compulsory risk assessment and temperature checks. In the three government hospitals (not the designated COVID-19 hospital), patients admitted for pneumonia and those with risk factors for COVID-19 were screened. In this paper, we describe this screening process from 9 March to 30 April 2020.

## Materials and methods

Screening for SARS-CoV-2 was implemented in the three government hospitals for all patients referred from clinics or who presented to the emergency departments for admission and who met the screening criteria. The standardized screening criteria were any of the following: community-acquired pneumonia (lower respiratory symptoms with no history of recent hospital admission), radiological changes consistent with pneumonia, previous quarantine within four weeks of contact with a confirmed COVID-19 case or travel to affected countries in the previous 14 days.

Patients were admitted to designated holding wards in each hospital, and nasopharyngeal swabs were taken and tested by reverse transcriptase polymerase chain reaction (RT–PCR) at the National Virology Reference Laboratory. Test results were usually available within 12 hours. While in the holding wards, patients continued to receive appropriate treatment and were screened for other infections, as indicated (dengue, malaria and various bacterial infections).

Patients who tested negative for SARS-CoV-2 were moved out of the holding wards to the main wards for continuation of care. Patients who tested positive were informed of their results, and transferred to the National Isolation Centre for further management. The Department of Public Health was informed of any positive results to initiate contact tracing without delay. Patients were interviewed according to the usual contact-tracing protocol, and family members and contacts were screened for SARS-CoV-2 with RT–PCR testing and quarantined for 14 days.

All positive SARS-CoV-2 cases, i.e. those detected by screening and those subsequently identified through contact tracing of cases, were transferred to the National Isolation Centre for treatment. Patients were admitted initially for a minimum of 14 days and were discharged only when they were symptom-free for three consecutive days and had two consecutive negative RT–PCR tests on days 12 and 14 of hospitalization. After discharge, patients were obliged to self-isolate for 14 days; a repeat swab was taken and tested on day 11 after discharge. Patients were considered cured once they had a negative swab and had completed 14 days of self-isolation. Patients who retested positive during self-isolation were readmitted for further management. Testing was repeated immediately, and the patients were discharged only after two consecutive negative swabs 24 hours apart. Our criteria have since changed and we no longer retest patients on day 11 after discharge. (*3*)

## Results

During the study period, 225 patients had been admitted to the holding wards in the three government hospitals. Most of the patients (90%) were admitted from a medical specialty: eight from surgical and 14 from renal specialties. Seven had been admitted to an intensive care unit and 35 to a high-dependency unit (**Table 1**).

**Table 1:** Number of admissions isolated and screened for COVID-19 by hospital and specialty, Brunei Darussalam, 9 March–30 April 2020

Specialty	Hospital 1		Hospital 2		Hospital 3	
	Holding ward 1	Holding ward 2 (high dependency)	Holding ward	Intensive care unit	Holding ward	Total
Medical	132	31	30	7	3	203
**Surgical**	8	0	-	-	-	8
**Renal**	10	4	-	-	-	14
**Total**	150	35	30	7	3	225

Of the 225 patients, only one (41-year-old man without comorbidities or travel history) was positive for SARS-CoV-2. This patient had presented five times to health-care services (four times to clinics and most recently to the emergency department of the main hospital) with fever and respiratory symptoms that had persisted despite symptomatic treatment. No contact with a possible or confirmed case was reported at any visit. After COVID-19 was confirmed, the contact history was reviewed, and the patient was linked to a confirmed case. The patient was immediately transferred to the National Isolation Centre for treatment. His course of illness was uncomplicated, and he was discharged after 15 days of hospitalization and two consecutive negative RT–PCR tests. A swab taken 11 days after discharge was negative. The 12 health-care workers involved in the care of this case at the original hospital were screened for SARS-CoV-2 and found to be negative.

Contact tracing for this case resulted in two additional COVID-19 cases: the patient’s spouse and daughter. Both were tested the day after the index case was diagnosed. The daughter, who already had mild fever and headache for two days, tested positive. The spouse, who was then presymptomatic, tested negative and was placed under a 14-day quarantine. She was retested 7 days later during quarantine when she developed sore throat and rhinorrhea, and was then positive. Both were admitted to the National Isolation Centre soon after testing positive (daughter two days and spouse eight days after diagnosis of index case) and were discharged after 14 (daughter) and 20 days (spouse). The spouse was readmitted for a further four days after retesting positive on day 11 after discharge.

## Discussion

Our experience highlights the importance of screening in hospitals during the COVID-19 outbreak. Although only one positive case was detected, we consider this programme a success, as, if the programme had not been carried out, nosocomial spread might have occurred. Nosocomial transmission has been reported, with significant consequences, including the deaths of health-care workers and other patients. (*4*-*7*) Hospitalized patients are usually older adults who have comorbidities that place them at higher risk for complications. (*8*) Screening for SARS-CoV-2 should therefore be maintained in health-care settings as the pandemic continues, with appropriate infection prevention and control (IPC) measures.

Our screening programme had implications not only for the hospitals but also for the community. Contact tracing for the case detected by screening led to the identification of two community cases, the patient’s spouse and daughter. They became mildly symptomatic during their illness; the daughter was symptomatic at first testing, and the spouse became symptomatic 7 days after initially testing negative and later retested positive. Their symptoms resolved without treatment during hospitalization. These two cases could have been missed if the index patient had not been diagnosed, with a potential risk for community spread. Detection of the initial case upon hospital admission and isolation of the two additional cases prevented further community spread.

The limitations encountered included the continuously changing criteria for SARS-CoV-2 in the earlier part of the pandemic, especially with respect to countries of travel. Initially, we categorized countries by risk categories according to the level of infection and presence of community spread; however, as more countries became affected, any travel history was considered a risk factor. The selection of patients for screening partially depended on the admitting doctors’ suspicion and interpretation of radiological changes. Even with set criteria, we relied on the vigilance and awareness of front-line workers of ever-changing guidelines and protocols. In addition, there will always be variation in doctors’ threshold for screening, and the screening yield was low (only one positive of 225 screened; 0.44% yield), especially among those with chronic pulmonary problems, such as chronic obstructive pulmonary disease and past tuberculosis, who had the expected radiological changes. Simple, non-infective exacerbations would have been identified during screening. Unnecessary isolation in the holding wards, even for a short time, can be detrimental to patients, particularly those who require intensive medical care. The strict IPC measures required in these wards further burdens patients and staff. Inappropriate admission to the holding wards also incurs costs, with inappropriate use of limited resources. We consider, however, that use of resources was acceptable, despite the low rate of detection, given that there was community spread in the country.

The areas that would improve the screening programme include: rapid dissemination and implementation of revised criteria and other relevant documents to front-line health-care workers; maintaining open communication among team members in various departments; and continuous audits of screened patients to improve the screening process.
